# Canalization of the evolutionary trajectory of the human influenza virus

**DOI:** 10.1186/1741-7007-10-38

**Published:** 2012-04-30

**Authors:** Trevor Bedford, Andrew Rambaut, Mercedes Pascual

**Affiliations:** 1 Department of Ecology and Evolutionary Biology, University of Michigan, Ann Arbor, MI, 48109, USA; 2 Howard Hughes Medical Institute, University of Michigan, Ann Arbor, MI, 48109, USA; 3 Institute of Evolutionary Biology, University of Edinburgh, Edinburgh, EH9 3JT, UK; 4 Fogarty International Center, National Institutes of Health, Bethesda, MD, 20892, USA

## Abstract

**Background:**

Since its emergence in 1968, influenza A (H3N2) has evolved extensively in genotype and antigenic phenotype. However, despite strong pressure to evolve away from human immunity and to diversify in antigenic phenotype, H3N2 influenza shows paradoxically limited genetic and antigenic diversity present at any one time. Here, we propose a simple model of antigenic evolution in the influenza virus that accounts for this apparent discrepancy.

**Results:**

In this model, antigenic phenotype is represented by a *N*-dimensional vector, and virus mutations perturb phenotype within this continuous Euclidean space. We implement this model in a large-scale individual-based simulation, and in doing so, we find a remarkable correspondence between model behavior and observed influenza dynamics. This model displays rapid evolution but low standing diversity and simultaneously accounts for the epidemiological, genetic, antigenic, and geographical patterns displayed by the virus. We find that evolution away from existing human immunity results in rapid population turnover in the influenza virus and that this population turnover occurs primarily along a single antigenic axis.

**Conclusions:**

Selective dynamics induce a canalized evolutionary trajectory, in which the evolutionary fate of the influenza population is surprisingly repeatable. In the model, the influenza population shows a 1- to 2-year timescale of repeatability, suggesting a window in which evolutionary dynamics could be, in theory, predictable.

## Background

Epidemic influenza is responsible for between 250,000 and 500,000 global deaths annually, with influenza A (and in particular, A/H3N2) having caused the bulk of human mortality and morbidity [1]. Influenza A (H3N2) has continually circulated within the human population since its introduction in 1968, exhibiting recurrent seasonal epidemics in temperate regions and less periodic transmission in the tropics. During this time, H3N2 influenza has continually evolved both genetically and antigenically. Most antigenic drift is thought to be driven by changes to epitopes in the hemagglutinin (HA) protein [2]. Phylogenetic analysis of the relationships among HA sequences has revealed a distinctive genealogical tree showing a single predominant trunk lineage and side branches that persist for only 1 to 5 years before going extinct [3]. This tree shape is indicative of serial replacement of strains over time; H3N2 influenza shows rapid evolution, but low standing genetic diversity.

This observation has remained puzzling from an epidemiological standpoint. Antigenic evolution occurs rapidly, and strong diversifying selection exists to escape from human immunity; why then do we see serial replacement of strains rather than continual genetic and antigenic diversification? Indeed, simple epidemiological models show explosive diversity of genotype and phenotype over time [4,5]. Previous work has sought model-based explanations of the limited diversity of influenza, relying on short-lived strain-transcending immunity [4,5], complex genotype-to-phenotype maps [6], or a limited repertoire of antigenic phenotypes [7].

Experimental characterization of antigenic phenotype is possible through the hemagglutination inhibition (HI) assay, which measures the cross-reactivity of HA from one virus strain to serum raised against another strain [8]. The results of many HI assays can be combined to yield a two-dimensional map, representing antigenic similarity and distance between strains as an easily visualized and quantified measure [9]. The path traced across this map by influenza A (H3N2) from 1968 until present is largely linear, showing serial replacement of one strain by another; there are no major bifurcations of antigenic phenotype [9].

Herein, we seek to simultaneously model the genetic and antigenic evolution of the influenza virus. We represent antigenic phenotypes as points in a *N*-dimensional Euclidean space. Based on the finding that a two-dimensional map adequately explains observed antigenic distance between strains [9], we begin with antigenic phenotypes as points on a plane, but relax this assumption later on in the analysis. After exposure to a virus, a host's risk of infection is proportional to the Euclidean distance between the infecting phenotype and the closest phenotype in the host's immune history. Mutations perturb antigenic phenotype, moving phenotype in a random radial direction and for a randomly distributed distance. We implemented this geometrical model in a large-scale individual-based simulation intended to directly model the antigenic map and genealogical tree of the global influenza population. The simulation includes multiple host populations with different seasonal forcing, hosts with complete immune histories of infection, and viruses with antigenic phenotypes. As the simulation proceeds, infections are tracked and a complete genealogy connecting virus samples is constructed. Results shown here are for simulations of 40 years of virus evolution in a population of 90 million hosts.

## Results and discussion

### Antigenic evolution and genealogical patterns

**Figure 1:**
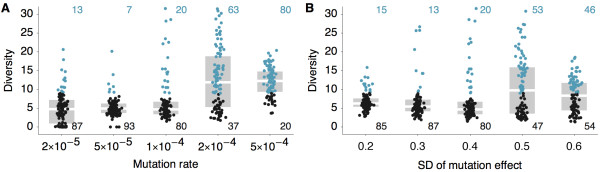
**Genealogical diversity at the end of 40 years across 100 simulations for varying mutational parameters**. Genealogical diversity varies with (**A**) mutation rate and with (**B**) standard deviation of mutation effect. Points represent individual simulation outcomes, and gray bars represent medians and interquartile ranges across replicate simulations. Outcomes with diversity greater than 9 years are shown in blue, and outcomes with diversity less than 9 years are shown in black. Counts of these two classes are shown in blue and black, respectively. Genealogical diversity is measured in years, mutation rate is measured in mutations per infection per day, and standard deviation of mutation effect is measured in antigenic units. Diversity less than 9 years is chosen as a cutoff based on observed patterns of branching in the H3N2 influenza genealogy.

The virus persists over the course of the 40-year simulation, and at the end of most simulations, there remain only a few closely related viral lineages, indicating that genealogical diversity is restricted by evolution in the two-dimensional antigenic landscape. Reduced diversity is substantially more common in models with less mutation or models with less variable mutation effects (Figure 1). At higher mutation rates, viruses may move apart in antigenic phenotype too rapidly for competition to always eliminate the weaker of two diverging lineages. Similarly, with high variance in mutational effect, there can sometimes emerge new antigenic types, too distant from the existing population to suffer limiting competitive pressure. Both these scenarios lead to coexistence of multiple antigenic phenotypes. We thus restrict the model to parameter regimes with lower mutation rates and lower mutation effect variances. We primarily focus on the model with 10^-4 ^mutations per infection per day and mutation effects with a standard deviation of 0.4 antigenic units. In this model, 80 out of the 100 replicate simulations show reduced genealogical diversity (defined as less than 9 years of evolution separating contemporaneous viruses). We conditioned the following analysis on these 80 simulations, compiling summary statistics across this pool and presenting a detailed analysis of a single representative simulation.

**Figure 2:**
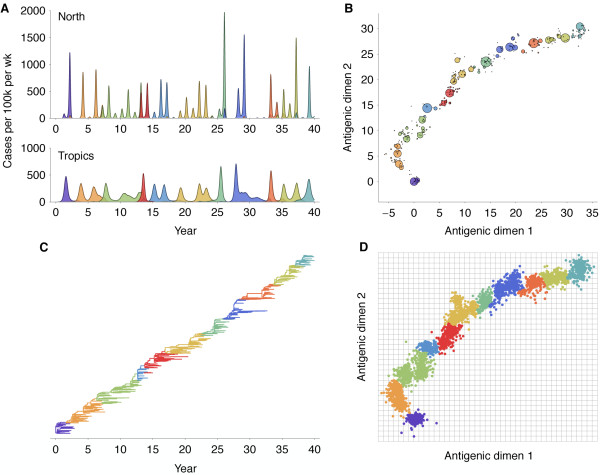
**Simulation results showing epidemiological, antigenic, and genealogical dynamics**. (**A**) Weekly time series of incidence of viral infection in north and tropics regions. (**B**) Two-dimensional antigenic phenotypes of 5,943 viruses sampled over the course of the simulation. Each discrete virus phenotype is shown as a bubble, with the bubble area proportional to the number of times this phenotype was sampled. (**C**) Genealogical tree depicting the infection history of 376 samples from the virus population. Parent/offspring relationships were tracked over the course of the simulation, giving a direct observation of the genealogy rather than a phylogenetic inference. (**D**) Antigenic map depicting phenotypes of 5,943 viruses sampled over the course of the simulation. To approximate experimental noise present in the observed antigenic map of H3N2 influenza, noise was added to each sample, and the resulting observations were grouped into 11 clusters and colored accordingly. Grid lines show single units of antigenic distance.

**Figure 3:**
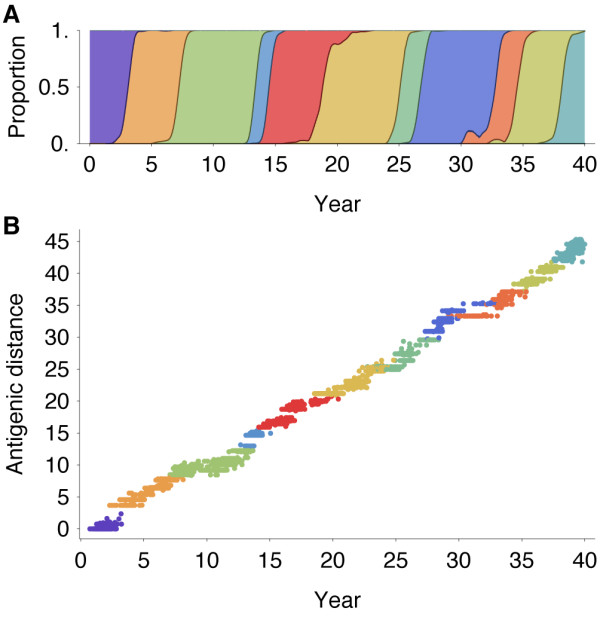
**Antigenic evolution over the course of the 40-year simulation**. (**A**) Proportion of virus population composed of each antigenic cluster through time. (**B**) Antigenic distance from the initial phenotype (*x *= 0, *y *= 0) for each of 5,943 virus samples relative to time of virus sampling. Viruses were sampled at a constant rate proportional to prevalence, and coloring was determined from the antigenic map in Figure 2D.

The model exhibits annual winter epidemics in temperate regions and less periodic epidemics in the tropics (Figure 2A). Across replicate simulations, we observe average yearly attack rates of 6.8% in temperate regions and rates of 7.1% in the tropics, comparable with estimated attack rates of influenza A (H3N2) of 3% to 8% per year [10,11]. Over the course of the simulation, the virus population evolves in antigenic phenotype exhibiting, at any point, a handful of highly abundant phenotypes sampled repeatedly and a large number of phenotypes appearing at low abundance (Figure 2B). The observed antigenic map of H3N2 influenza includes substantial experimental noise; replicate strains appear in diverse positions on the observed map. By including measurement noise on antigenic locations (see Methods), we approximate an experimental antigenic map of H3N2 influenza (Figure 2D). Over the 40-year simulation, antigenic drift moves the virus population at an average rate across replicate simulations of 1.05 antigenic units per year, corresponding closely to the empirical rate of 1.2 units per year [9]. The appearance of clusters in the antigenic map comes from the regular spacing of high abundance phenotypes combined with measurement noise. Over time, clusters of antigenically similar strains are replaced by novel clusters of more advanced strains (Figure 3A). Across replicate simulations, clusters persist for an average of 5.0 years, measured as the time it takes for a new cluster to reach 10% frequency, peak, and decline to 10% frequency. The transition between clusters occurs quickly, taking an average of 1.8 years.

Remarkably, although antigenic phenotype is free to mutate in any direction in the two-dimensional space, selection pressures force the virus population to move in nearly a straight line in antigenic space (Figure 2B). Across replicate simulations, 94% of the variance of antigenic phenotype can be explained by a single dimension of variation. This mirrors the empirical results showing a largely linear antigenic map for H3N2 influenza isolates from 1968 to 2003 [9]. Because of the primarily one-dimensional movement, antigenic distance from the original phenotype increases nearly linearly with time (Figure 3B). Antigenic evolution occurs in a punctuated fashion; periods of relative stasis are interspersed with more rapid antigenic change (Figure 3B). Antigenic and epidemiological dynamics show a fundamental linkage so that large jumps of antigenic phenotype result in increased rates of infection (Figure 2).

The genealogical tree connecting the evolving virus population appears characteristically sparse with pronounced trunk and short-lived side branches (Figure 2C). This tree shape is reflected in low levels of standing diversity; across replicates, an average of 5.68 years of evolution separates two randomly sampled viruses in the population. This result matches well with the average diversity observed in influenza A (H3N2) of 5.65 years, separating randomly sampled contemporaneous viruses [12]. A spindly genealogical tree is indicative of population turnover, wherein novel antigenic phenotypes continually replace more primitive 'spent' phenotypes, purging their genealogical diversity. In general, natural selection reduces effective population size and genealogical diversity [12].

**Figure 4:**
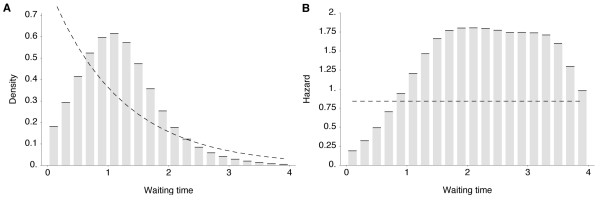
**Observed vs. expected distributions of waiting times between phenotypic mutations along genealogy trunk**. (**A**) Histogram bins show the observed distribution of waiting times in years across 80 replicate simulations representing 1,584 mutations. The mean of this distribution is 1.76 years. The dashed line shows the Poisson process expectation of exponentially distributed waiting times. (**B**) The density distribution of waiting times is transformed into a hazard function, representing the rate of trunk mutation after a specific waiting time. The dashed line shows the memoryless hazard function of the Poisson process expectation.

Selective pressures can be examined by comparing which mutations fix, that is, incorporated into the progenitor trunk lineage, and which mutations are lost, that is, incorporated into side branches bound for extinction. This approach has shown that, in influenza A (H3N2), natural selection promotes mutations to epitope sites in the HA1 region [13,14]. By examining antigenic mutations, we find a corresponding effect in simulated evolutionary trajectories (Table 1). Additionally, we find that trunk mutations occur at strikingly regular intervals, with less variation of waiting times than expected under a simple random process (Figure 4). There is a relative scarcity of mutation events occurring in intervals under 1 year and a relative excess of mutation events occurring in 2- to 3-year intervals (Figure 4). This pattern arises from clonal interference between competing mutations which reduces variability in the fixation process of adaptive substitutions [15].

**Table 1:** Rates of mutation and phenotypic change on trunk and side branches and mutational expectation

	Baseline	Side branch	Trunk	Trunk/side branch
Mutation size (antigenic units)	0.60	0.79	1.58	1.99×
Mutation rate (mutations per year)	0.04	0.06	0.81	13.23×
Antigenic flux (antigenic units per year)	0.02	0.05	1.27	26.25×

### Spatial dynamics

**Figure 5:**
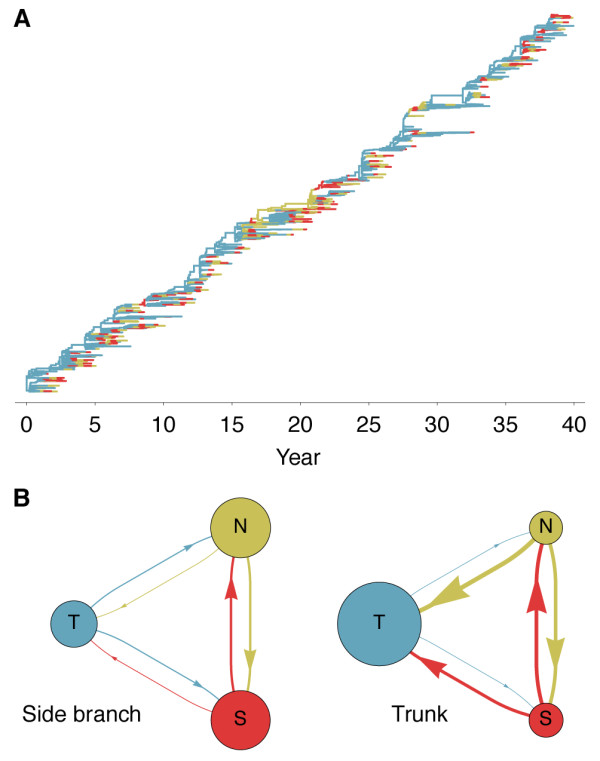
**Patterns of geographic movement of virus lineages**. (**A**) Evolutionary relationships among 376 viruses sampled evenly through time colored by geographic location. Lineages residing in the north (N), south (S), and tropics (T) are colored yellow, red, and blue, respectively. (**B**) Observed migration rates between regions on side branch lineages (left) and on trunk lineages (right). Arrows denote movement of lineages, and arrow width is proportional to migration rate. Circle area is proportional to the expected stationary frequency of a region given the observed migration rates. In both cases, migration rates are calculated across 80 replicate simulations.

The genealogical tree also contains detailed information on the history of migration between regions. We find that, consistent with empirical estimates [16,17], the trunk resides primarily within the tropics, where seasonal dynamics are less prevalent (Figure 5A). Across replicate simulations, we observe 72% of the trunk's history within the tropics and 28% within temperate regions. With symmetric host contact rates and equal host population sizes, and without seasonal forcing, we would expect trunk proportions of one third for each region. We calculated rates of migration based on observed event counts across replicate simulations, separating region-specific rates on side branches from region-specific rates on trunk branches. We find that migration patterns on side branches are close to symmetric, with similar rates between all regions, while migration patterns on trunk branches are highly asymmetric, with high rates of movement between temperate regions and from temperate regions into the tropics (Figure 5B). Extrapolating from these rates, we arrive at an expected stationary distribution of trunk location of 76% tropics and 24% temperate regions, in line with the observed residency patterns of the trunk. It may at first seem counterintuitive to see higher rates of movement from the temperate regions into the tropics along trunk branches, but it makes sense when thought of in terms of conditional probability. Only those lineages that remain in the tropics, migrate into the tropics, or which rapidly migrate between the north and south have a chance at becoming the trunk lineage, while lineages that remain within the temperate regions are doomed to extinction. Along similar lines, Adams and McHardy [18] use a modeling approach to show the importance of nonseasonal transmission to the evolution of the virus.

These findings suggest that persistence and migration are fundamentally connected and have important implications for future phylogeographic analyses. Russell *et al. *[16] emphasize a source-sink model of movement of the HA protein of influenza A (H3N2) based on their finding of a trunk lineage residing within China and the Southeast Asian tropics, whereas Bedford *et al. *[17] emphasize a global metapopulation model based on phylogenetic inference of migration rates across the entire tree. Our results suggest that both scenarios are simultaneously possible; side branches may be highly volatile, moving rapidly and symmetrically between regions, while the trunk lineage may be more stable remaining within a region (or within a highly connected network of regions) that has more persistent transmission. In light of these results, we suggest that future work on the phylogeography of influenza take into account trunk vs. side branch differences in migration patterns.

### Correspondence between model and data

In our model, antigenic evolution is driven by the appearance of novel antigenic variants that best escape existing human immunity. Although multiple epidemiological/evolutionary mechanisms have been proposed to explain the restricted genetic diversity and rapid population turnover of influenza A (H3N2) [4-7], our results show that a simple model coupling antigenic and genealogical evolution exhibits broad explanatory power. We find a strong correspondence between the antigenic and genealogical patterns generated by our model (Figure 2) and patterns of genetic and antigenic evolution exhibited by influenza A (H3N2) [3,9]. Our model simultaneously captures seasonal attack rates, the rate and pattern of antigenic drift, genealogical diversity, and geographic migration patterns.

Our model predicts that detailed classification of influenza strains will support a relatively small number of predominant phenotypes. Rather than each influenza strain possessing a unique antigenic location, many strains group together with shared antigenic phenotypes (Figure 2B). We suggest that a large proportion of intra-cluster variation in the observed antigenic map is due to experimental noise rather than each strain possessing a unique antigenic location. The relationship between Figure 2B,D illustrates this effect, where a large number of antigenic locations emerge from a comparatively small number of unique antigenic phenotypes. Additionally, our model accurately predicts the contrasting dynamics of other types/subtypes of influenza. We find that lowering mutation size/effect or lowering intrinsic *R*_0 _results in decreased incidence, slower antigenic movement, and greater genealogical diversity, all distinguishing characteristics of H1N1 influenza and influenza B [see Figure S1 in Additional file 1].

The historical record of influenza evolution suggests that bifurcation of viral lineages is rare, but possible. We have observed no bifurcations in H1N1 influenza from 1918 to 1957 and again from 1977 to 2010, no bifurcations in H2N2 influenza from 1957 to 1968, and no bifurcations of H3N2 influenza from 1968 to 2010. We have observed one bifurcation in influenza B from 1940 to 2010. Thus, ignoring differences between influenza types and subtypes, we have very roughly observed a rate of one major bifurcation in 195 years of evolution. In our model, in 20 out of 100 replicate simulations, we observe a deep bifurcation in the viral genealogy, which translates to observing one deep bifurcation in 200 years of evolution. Thus, we suggest that the 20 of 100 simulations where deep branching occurs are not necessarily evidence of poor model t. Similar to Koelle *et al. *[19], we assume that although the historical evolution of H3N2 influenza followed the path of a single lineage, it could have included a major bifurcation.

In our model, when antigenic phenotype remains static, there may be multiple consecutive seasons without appreciable incidence (Figure 2A), a pattern apparently absent from H3N2 influenza [20]. Additionally, we observe antigenic trajectories that are more linear and deterministic than the highly clustered trajectory observed by Smith *et al. *[9]. We suggest that any model exhibiting punctuated evolution broadly consistent with the punctuated change seen in the experimental antigenic map will show similar patterns of incidence. We can 'fix' the incidence patterns but at the cost of too smooth an antigenic map [see Figure S2 in Additional file 1]. Evolutionary patterns of the neuraminidase (NA) protein may provide an explanation. Epitopes in the HA and NA proteins are jointly responsible for determining antigenicity [2], and it is now clear that levels of adaptive evolution are similar between HA and NA [21]. Thus, changes in NA may be driving incidence patterns as well, resulting in an observed time series of incidence partially divorced from the antigenic map of HA. Incorporating antigenic evolution of NA could thus yield a rougher antigenic map for HA, more closely matching experimental results, while simultaneously yielding smoother year-to-year incidence patterns.

It remains a central question as to the extent that short-lived strain-transcending immunity is responsible for influenza's limited diversity and spindly genealogical tree [4,5]. Our findings suggest a possible resolution. Although lacking short-lived immunity, our model shows a detailed correspondence to both the antigenic map and genealogical tree of H3N2 influenza. If an antigenic map were to show a deep bifurcation, where two viral lineages move in different antigenic directions, then we would expect the same bifurcation to be evident in the genealogical tree. Short-lived strain-transcending immunity provides a mechanism by which lineages may diverge in antigenic phenotype but still show epidemiological interference. This mechanism would explain a situation where bifurcations emerge in the antigenic map, but competition results in the extinction of divergent antigenic lineages. In our model, one cluster leads to another cluster in orderly succession, and there is never competition between antigenically distant clusters. Thus, short-lived strain-transcending immunity is not required to limit diversity in the model. This is not to say that short-lived strain-transcending immunity is not present; observed interference between subtypes [4,22], evolution at CTL epitopes [23], and the exclusion of the Beijing/89 cluster by the antigenically distant Beijing/92 cluster [9] all suggest some form of more general interaction between influenza viruses.

### Linear antigenic movement

**Figure 6:**
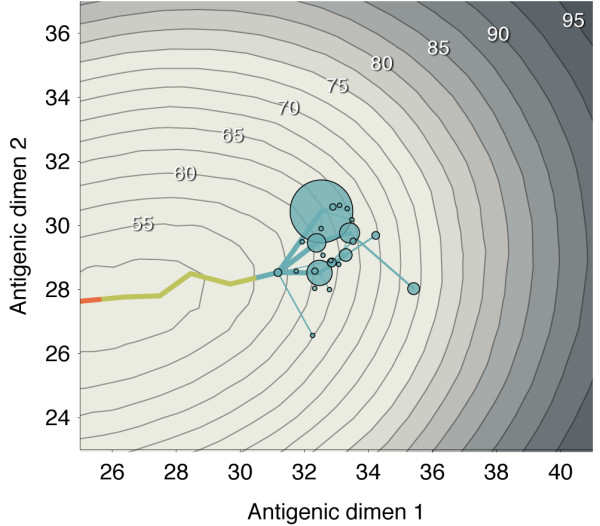
**Host immunity and antigenic history of the virus population**. Contour lines represent the state of host immunity at the end of the 40-year simulation. They show the mean risk of infection (as a percentage) after a random host in the population encounters a virus bearing a particular antigenic phenotype. Contour lines are spaced in intervals of 2.5%. Bubbles represent a sample of antigenic phenotypes present at the end of the 40-year simulation. The area of each bubble is proportional to the number of samples with this phenotype. Lines leading into these bubbles show past antigenic history. The current phenotypes rapidly coalesce to a trunk phenotype. The movement of the virus population from the left to the center of the figure can be seen from the antigenic history. At the end of the simulation, several virus phenotypes exist with similar antigenic locations; all of these phenotypes lie significantly ahead of the peak of host immunity.

It might seem reasonable for one viral lineage to move in one antigenic direction, while another lineage moves tangentially, eventually resulting in two non-interacting viral lineages. Instead, we find that movement in a single antigenic direction is favored, resulting in most replicate simulations showing low standing diversity (Figure 1). The origins of this pattern can be seen in the interaction between virus evolution and host immunity (Figure 6). As the virus population evolves forward, it leaves a wake of immunity in the host population, and evolution away from this immunity results in the canalization of the antigenic phenotype; mutations that continue along the line of primary antigenic variation will show a transmission advantage compared to more tangential mutations.

Following the work of Smith *et al. *[9], it remained an open question of why a two-dimensional map should explain the antigenic variation of H3N2 influenza, although the authors astutely speculated that 'there is a selective advantage for clusters that move away linearly from previous clusters as they most effectively escape existing population-level immunity, and this is a plausible explanation for the somewhat linear antigenic evolution in regions of the antigenic map.' This hypothesis remained to be tested. Here, we show from a simple model of epidemiology and evolution that a linear trajectory of antigenic evolution dynamically emerges due to basic selective pressures. This result simultaneously explains the linear pattern of antigenic drift [9] and the characteristically spindly genealogical tree [3] exhibited by influenza A (H3N2).

For this process to take hold, the virus population needs to be somewhat mutationally limited; if functional antigenic variants of novel phenotype emerge too quickly, then antigenic change will occur too rapidly for competition to winnow down the virus population to a single lineage (Figure 1). Assuming that antigenic mutations have an average effect of 0.6 antigenic units and a standard deviation of 0.4 units, then the rate of new antigenic mutations cannot be greater than approximately 10^-4 ^mutations per day (Figure 1). Thus, it is important that the rate of 10^-4 ^mutations per day be biological plausible. Here, we take the rate of synonymous substitution as a proxy for the neutral rate of mutation. The rate of synonymous change has been estimated at 2.5 × 10^-6 ^per site per day [24]. As there are approximately two nonsynonymous sites per codon in influenza [25], this gives a neutral rate of amino acid change of approximately 5 × 10^-6 ^per site per day. Other work has shown that there appear to be approximately 18 amino acid sites implicated in the majority of adaptive change [13]. These sites evolve along the trunk of the phylogeny at rate of 0.053 substitutions per site per year or at a combined rate of 0.95 substitutions per year [4]. This result agrees well with our finding of 0.81 antigenic mutations per year on the phylogeny trunk (Table 1). If we assume 18 sites involved in antigenic change, this gives an overall rate of antigenic mutation of 9 × 10^-5 ^per day. Thus, we believe that 10^-4 ^mutations per day represents a biologically reasonable estimate.

To consider to what extent these results were contingent on the dimensionality of the underlying antigenic model, we further implemented our model in a 10-dimensional antigenic space. Here, mutations occur as 10-spheres, but the distance moved by a mutation is the same as in the previous two-dimensional formulation. We arrive at nearly the same results with this model; principal components analysis shows that the first and second dimensions of variation account for 87% and 7%, respectively, of the total variance [see Figure S3 in Additional file 1]. Thus, our model predicts that future work probing mutational effects will support an underlying high-dimensional antigenic space, even though a two-dimensional map is sufficient to explain observed antigenic relationships among evolving strains.

### Winding back the tape

The 40-year simulation of influenza dynamics shows broad correspondence with observed patterns. However, year-to-year details are not captured, for example, years that undergo antigenic transitions in the 40-year simulation do not match up with observed years of antigenic transitions. Over long time spans, year-to-year correspondence seems impossible to achieve in this sort of stochastic system, where evolution is often driven by chance mutations of large antigenic effect. However, correspondence in the shorter term may be possible. To test this, we examined repeatability in replicate simulations, showing what happens when we 'wind back the tape' [26] on the evolution of the virus. We ran 100 replicate simulations, each starting from the endpoint of the representative 40-year simulation shown in Figure 2. The starting point for these replicate simulations was the exact end state of the 40-year simulation, including the frequencies of every virus strain and the entire host immune profile. These replicate simulations were run for an additional 6 years, and all evolutionary and epidemiological parameters were identical to the initial 40-year simulation.

**Figure 7:**
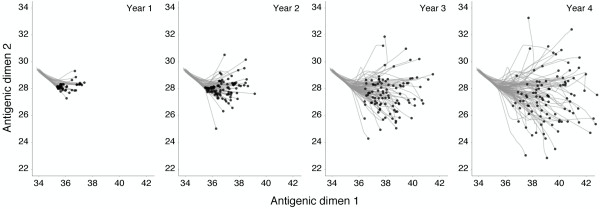
**Antigenic phenotypes over the course of 4 years of evolution across 100 replicate simulations starting from identical initial conditions**. Replicate simulations were initialized with the end state of the 40-year simulation shown in Figure 2. Each panel shows an additional year of evolution, with black points representing the mean antigenic phenotypes of the 100 replicate simulations and gray lines representing the history of each mean antigenic phenotype.

**Figure 8:**
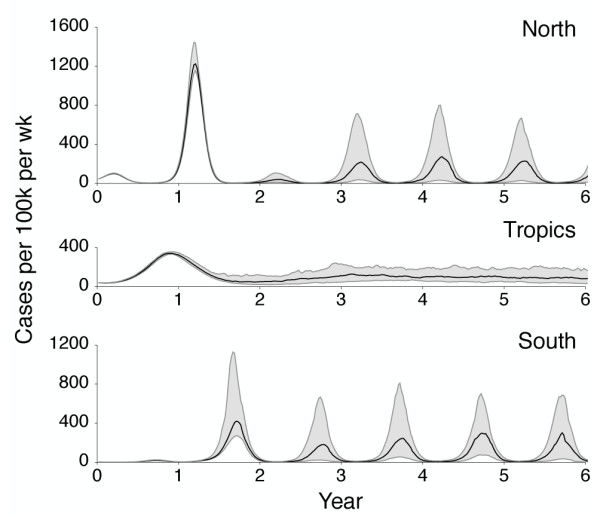
**Time series of incidence across 100 replicate simulations with identical initial conditions**. Panels show incidence in the north, tropics, and south regions over the course of 6 years. Solid black lines represent the median weekly incidence across the 100 replicate simulations, while gray intervals represent the interquartile range across simulations. There is little variability for the first year of replicate simulations. Replicate simulations were initialized with the end state of the 40-year simulation shown in Figure 2.

Initially, we find a great detail of repeatability; during the first year of evolution, every replicate virus population undergoes a similar antigenic transition (Figure 7), resulting in a repeatable peak in northern hemisphere incidence (Figure 8). After 3 years, repeatability has mostly disappeared, with antigenic phenotype and incidence appearing highly variable across replicates (Figures 7 and 8). The 1- to 2-year timescale of repeatability can be explained by the presence of standing antigenic variation. In the initial virus population, there are several novel antigenic variants present at low frequency (Figure 6), one of which, without fail, comes to predominate the virus population.

We see that the initial evolutionary trajectory, during which time standing variation plays out, is highly repeatable and thus predictable given enough information and the right methods of analysis. However, prediction of longer-term evolutionary scenarios will necessarily be difficult or impossible except in a vague sense. Through careful surveillance e orts and genetic and antigenic characterization of influenza strains, the World Health Organization makes twice-yearly vaccine strain recommendations [27]. It should be possible to combine these sorts of modeling approaches with surveillance data to gauge the likelihood that a sampled variant will spread through the population.

## Conclusions

Recent work on empirical fitness landscapes has shown that natural selection follows few mutational paths [28]. The spindly genealogical tree and the almost linear serial replacement of influenza strains have remained a puzzling phenomenon. We suggest that the evolutionary and epidemiological dynamics displayed by the influenza virus may simply be explained as an outgrowth of selection to avoid host immunity. Natural selection can only 'see' one step ahead and so sacrifices long-term gains for short-term advantages. The result is a canalized evolutionary trajectory lacking antigenic diversification.

## Methods

### Transmission model

To characterize the joint epidemiological, genealogical, antigenic, and spatial patterns of influenza, we implemented a large-scale individual-based model (full simulation source code available at http://www.trevorbedford.com/antigen/). This model consists of daily time steps, in which the states of hosts and viruses are updated. Hosts may be born, may die, may contact other hosts allowing viral transmission, or may recover from infection. Viruses may mutate in antigenic phenotype. Each simulation ran for 40 years of model time.

Hosts in this model are divided between three regions: north, south, and tropics. There are 30 million hosts within each of the three regions, giving N = 9 × 10^7 ^hosts. Host population size remains fixed at this number, but vital dynamics cause births and deaths of hosts at a rate of 1/30 years = 9.1 × 10^-5 ^per host per day. Within each region, transmission proceeds through mass action with contacts between hosts occurring at a rate of *β *= 0.36 per host per day. Regional transmission rates in temperate regions vary according to sinusoidal seasonal forcing with amplitude *∊ *= 0.15 and opposite phase in the north and in the south. Transmission rate does not vary over time in the tropics. Transmission between region *i *and region *j *occurs at rate *mβ_i_*, where *m *= 0.001 and is the same between each pair of regions and *β_i _*is the within-region contact rate. Hosts recover from infection at rate *v *= 0.2 per host per day so that *R*_0 _in a naive host population is 1.8. An individual cannot be simultaneously infected with multiple virus lineages; there is no superinfection in the model.

Each virus possesses an antigenic phenotype, represented as a location in Euclidean space. Here, we primarily use a two-dimensional antigenic location. After recovery, a host 'remembers' the phenotype of its infecting virus as part of its immune history. When a contact event occurs and a virus attempts to infect a host, the Euclidean distance from the infecting phenotype *ϕ_v _*is calculated to each of the phenotypes in the host immune history $\phi_{h_{1}},\ldots ,\phi_{h_{n}}$. Here, 1 unit of antigenic distance is designed to correspond to a twofold dilution of antiserum in a HI assay [9]. The probability that infection occurs after exposure is proportional to the distance *d *to the closest phenotype in the host immune history. Risk of infection follows the form *ρ *= min{*d s*, 1}, where *s *= 0.07. Cross-immunity *σ *equals 1 - *ρ*. With no initial immunity in the host population, the virus undergoes a severe trough in prevalence after the initial pandemic increase. With this number of host individuals, the virus population usually stochastically dies out during this trough. To prevent this, we gave the initial host population enough immunity to slow down the initial virus upswing and place the dynamics closer to their equilibrium state; initial *R *was 1.28. Future work should attempt to more accurately model initial evolutionary dynamics.

As in the current analysis, previous studies [4,6,7] have assumed that host immune response is dictated by the closest phenotype in the immune history. Due to original antigenic sin, other phenotypes in the host immune history may have a disproportionate effect on host immunity. This may be an important aspect to modeling influenza and should be addressed in future studies. Our model follows that of Lin *et al. *[29] and Gog and Grenfell [30] in representing antigenic distance as the distance between points in a geometric space. By forcing one dimension to directly modulate *β*, Gog and Grenfell find an orderly replacement of strains. Kryazhimskiy *et al. *[31] use a two-dimensional strain space, in which cross-immunity between two strains is proportional to their distance in one dimension or the other, whichever is closer. This cross-immunity kernel directly favors moving along a diagonal line away from previous strains. Our model more closely approximates how HI distance is incorporated into the antigenic map of Smith *et al. *[9]; HI between strains is projected as a Euclidean distance, rather than as the closest distance between strains in either dimension one or two.

The initial virus population consisted of 10 infections each with the identical antigenic phenotype of {0, 0}. Over time, viruses evolve in antigenic phenotype. Each day, there is a chance *μ *= 10^-4 ^that an infection mutates to a new phenotype. This mutation rate represents a phenotypic rate, rather than genetic mutation rate, and can be thought of as arising from multiple genetic sources. When a mutation occurs, the virus's phenotype is moved in a random radial direction. Mutation size is sampled from a gamma distribution, with distribution parameters chosen to give a mean mutation size of δ_avg _= 0.6 units and a standard deviation of δ_sd _= 0.4 units. This distribution is parameterized so that mutation usually has little effect on antigenic phenotype but occasionally has a larger effect. This is similar to the neutral networks implemented by Koelle *et al. *[6], wherein most amino acid changes result in little decrease to cross-immunity between strains, but some changes result in large jumps in cross-immunity. This mutation model implicitly assumes independence between mutations; each mutation's effect is independent of genetic background.

### Model output

Daily incidence and prevalence are recorded for each region. During the course of the simulation, samples of current infections are taken from the evolving virus population at a rate proportional to prevalence. Each viral infection is assigned a unique identification (ID), and in addition, infections have their phenotypes, locations, and dates of infection recorded. In this model, viruses lack sequences and so standard phylogenetic inference of the evolutionary relationships among strains is impossible. Instead, the viral genealogy is directly recorded. This is made possible by tracking transmission events connecting infections during the simulation; infections record the ID of their 'parent' infection. Proceeding from a sample of infections, their genealogical history can be reconstructed by following consecutive links to parental infections. Following this procedure, lineages coalesce to the ancestral lineages shared by the sampled infections, eventually arriving at the initial infection introduced at the beginning of the simulation. Commonly, phylodynamic simulations generate sequences that are subsequently analyzed with a phylogenetic software to produce an estimated genealogy [4,6,32]. This step of phylogenetic inference is imperfect and computationally intensive, and by side-stepping phylogenetic reconstruction, we arrive at genealogies quickly and accurately. Other authors have implemented similar tracking of infection trees [33,34]. This genealogy-centric approach makes many otherwise difficult calculations transparent, such as calculating lineage-specific region-specific migration rates (Figure 5) and lineage-specific mutation effects (Table 1).

Infections are sampled at a rate designed to give approximately 6,000 samples over the course of the simulation, with genealogies constructed from a subsample of approximately 300 samples. The results presented in Figure 2 represent a single representative model output; 100 replicate simulations were conducted to arrive at statistical estimates.

### Parameter selection and sensitivity analysis

Estimating what the basic reproductive number *R*_0 _for seasonal influenza would be in a naive population is notoriously difficult. Season-to-season estimates of effective reproductive number *R *for the USA and France gathered from mortality time series display an interquartile range of 0.9 to 1.8 [35]. Geographic spread within the USA suggests an average seasonal *R *of 1.35 [36]. These estimates of *R *will be lower than the *R*_0 _of influenza due to the effects of human immunity. We assumed a *R*_0 _of 1.8, consistent with the upper range of seasonal estimates. Duration of infection was chosen based on patterns of viral shedding shown during challenge studies [37]. The linear form of the risk of infection and its increase as a function of antigenic distance *s *= 0.07 were based on experimental work on equine influenza [38] and from studies of vaccine effectiveness [39]. Between-region contact rate *m *was chosen to yield a trunk lineage that resides predominantly in the tropics. With much higher rates of mixing, the trunk lineage ceases to show a preference in the tropics, and with much lower rates of mixing, particular seasons in the north and the south will often be skipped. The amplitude of seasonal forcing *ε *was chosen to be just large enough to get consistent fade-outs in the summer months and is consistent with empirical estimates [40].

Mutational parameters were based, in part, on model behavior. We assumed 10 amino acid sites involved in antigenicity, each mutating at a rate of 10^-5 ^[41] to give a phenotypic mutation rate *μ *= 10^-4^per infection per day. We chose mutational effect parameters (*δ*_avg _= 0.6, *δ*_sd _= 0.4) that would give suitably fast rates of antigenic evolution corresponding to approximately 1.2 units of antigenic change per year while simultaneously giving clustered patterns of antigenic evolution [9]. Similar outcomes are possible under a variety of parameterizations. If mutations are more common (*μ *= 3 × 10^-4^) and show less variation in effect size (*δ*_avg _= 0.6, *δ*_sd _= 0.2), then antigenic drift occurs in a more continuous fashion, resulting in less variation in seasonal incidence and a smoother distribution of antigenic phenotypes [see Figure S2A,C in Additional file 1]. If mutations are less common (*μ *= 5 × 10^-5^) and show more variance in effect (*δ*_avg _= 0.7, *δ*_sd _= 0.5), then antigenic change occurs in a more punctuated fashion [see Figure S2B,D in Additional file 1]. Basic reproductive number *R*_0 _can be traded off with mutational parameters to some extent. Less mutational input and higher *R*_0 _will give similar patterns of antigenic drift and seasonal incidence. Similarly, Kucharski and Gog [42] find that increasing *R*_0 _results in increased rates of emergence of antigenically novel strains.

### Antigenic map

Antigenic phenotypes are modeled as discrete entities on the Euclidean plane; multiple samples have the same antigenic location. However, in the empirical antigenic map of influenza A (H3N2), each strain appears in a unique location [9]. We would argue that some of this pattern comes from experimental noise. Indeed, Smith *et al. *[9] find that observed measurements and measurements predicted from the map differ by an average of 0.83 antigenic units with a standard deviation of 0.67 antigenic units. We take this as a proxy for experimental noise and add jitter to each sampled antigenic phenotype by moving it in a random direction for an exponentially distributed distance with mean of 0.53 antigenic units. If two samples with the same underlying antigenic phenotype are jittered in this fashion, the distance between them averages 0.83 antigenic units with a standard deviation of 0.64 units.

We added noise to each of the 5,943 sampled viruses in this fashion, resulting in an approximated antigenic map (Figure 2D). Virus samples were then clustered following standard clustering algorithms. We tried clustering by the *k*-means algorithm and also agglomerative hierarchical clustering with a variety of linkage criterion. We found that clustering by Ward's criterion consistently outperformed other methods when measured in terms of within-cluster and between-cluster variances. However, the exact clustering algorithm had little effect on our overall results.

## Competing interests

The authors declare that they have no competing interests.

## Authors' contributions

TB and MP conceived the project. TB conducted the simulation experiments and performed statistical analyses. TB, AR, and MP analyzed the results and wrote the paper. All authors have read and approved the final manuscript.

## Authors' information

TB holds a Long-Term Fellowship from the European Molecular Biology Organization. AR works as a part of the Interdisciplinary Centre for Human and Avian Influenza Research (ICHAIR) and the University of Edinburgh's Centre for Immunity, Infection and Evolution (CIIE). MP is an investigator of the Howard Hughes Medical Institute.

## Supplementary Material

Additional file 1 **Supplementary figures**. Includes supplementary figures 1 to 3 detailing results from simulations with alternative parameter values. Click here for file
