# Supplementary material for: Canalization of the evolutionary trajectory of the human influenza virus
Source: BMC Biol. 2012 Apr 30;10:38. doi: 10.1186/1741-7007-10-38 (PMC3373370; doi:10.1186/1741-7007-10-38)
Supplement: Additional file 1 — Supplementary figures. Includes supplementary figures 1 to 3 detailing results from simulations with alternative parameter values. [file 1741-7007-10-38-S1.PDF]

Supporting Figures: Canalization of the evolutionary trajectory of the human influenza virus

Citation: Bedford T, Rambaut A, Pascual M. 2012. Canalization of the evolutionary trajectory of the human influenza virus. BMC Biology.

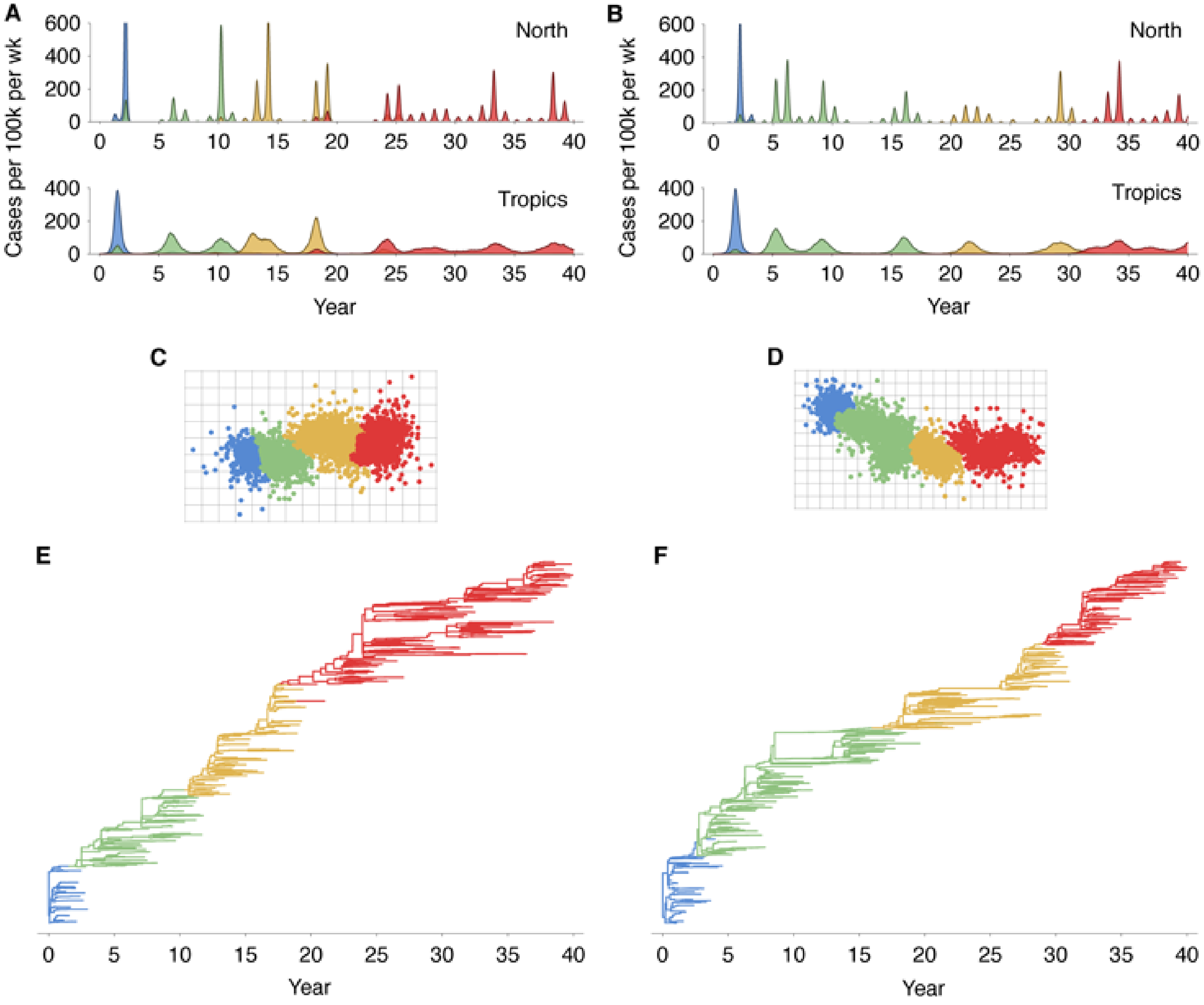

Figure S1. Simulation results showing epidemiological, antigenic and genealogical dynamics with weaker mutation ($\mu = 5 \times 10^{-5}$, $\delta_{avg} = 0.42$, $\delta_{sd} = 0.28$) or lower contact rate ($R_0 = 1.5$). (A) and (B) Weekly timeseries of incidence of viral infection in north and tropics regions for weaker mutation (A) and lower R_0 (B). (C) and (D) Antigenic map depicting phenotypes of viruses sampled over the course of the simulation for weaker mutation (C) and lower R_0 (D). Grid lines show single units of antigenic distance. (E) and (F) Genealogical tree depicting the infection history of samples from the virus population for weaker mutation (E) and lower R_0 (F).

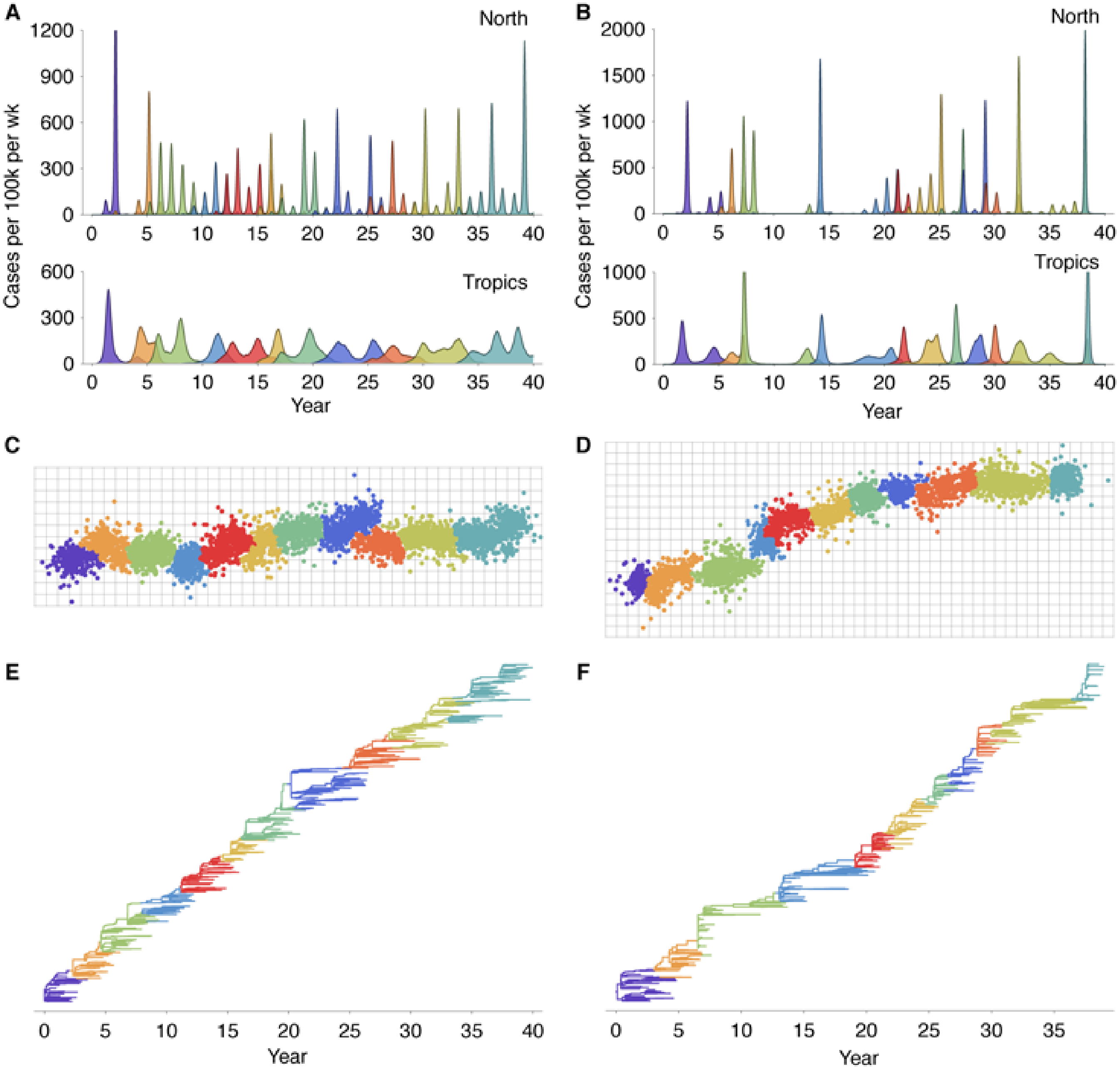

Figure S2. Simulation results showing epidemiological, antigenic and genealogical dynamics for 'smoother' mutation model ($\mu = 3 \times 10^{-4}$, $\delta_{avg} = 0.6$, $\delta_{sd} = 0.2$) and 'rougher' mutation model ($\mu = 5 \times 10^{-5}$, $\delta_{avg} = 0.7$, $\delta_{sd} = 0.5$). (A) and (B) Weekly timeseries of incidence of viral infection in north and tropics regions for 'smoother' mutation (A) and 'rougher' mutation (B). (C) and (D) Antigenic map depicting phenotypes of viruses sampled over the course of the simulation for 'smoother' mutation (C) and 'rougher' mutation (D). Grid lines show single units of antigenic distance. (E) and (F) Genealogical tree depicting the infection history of samples from the virus population for 'smoother' mutation (E) and 'rougher' mutation (F).

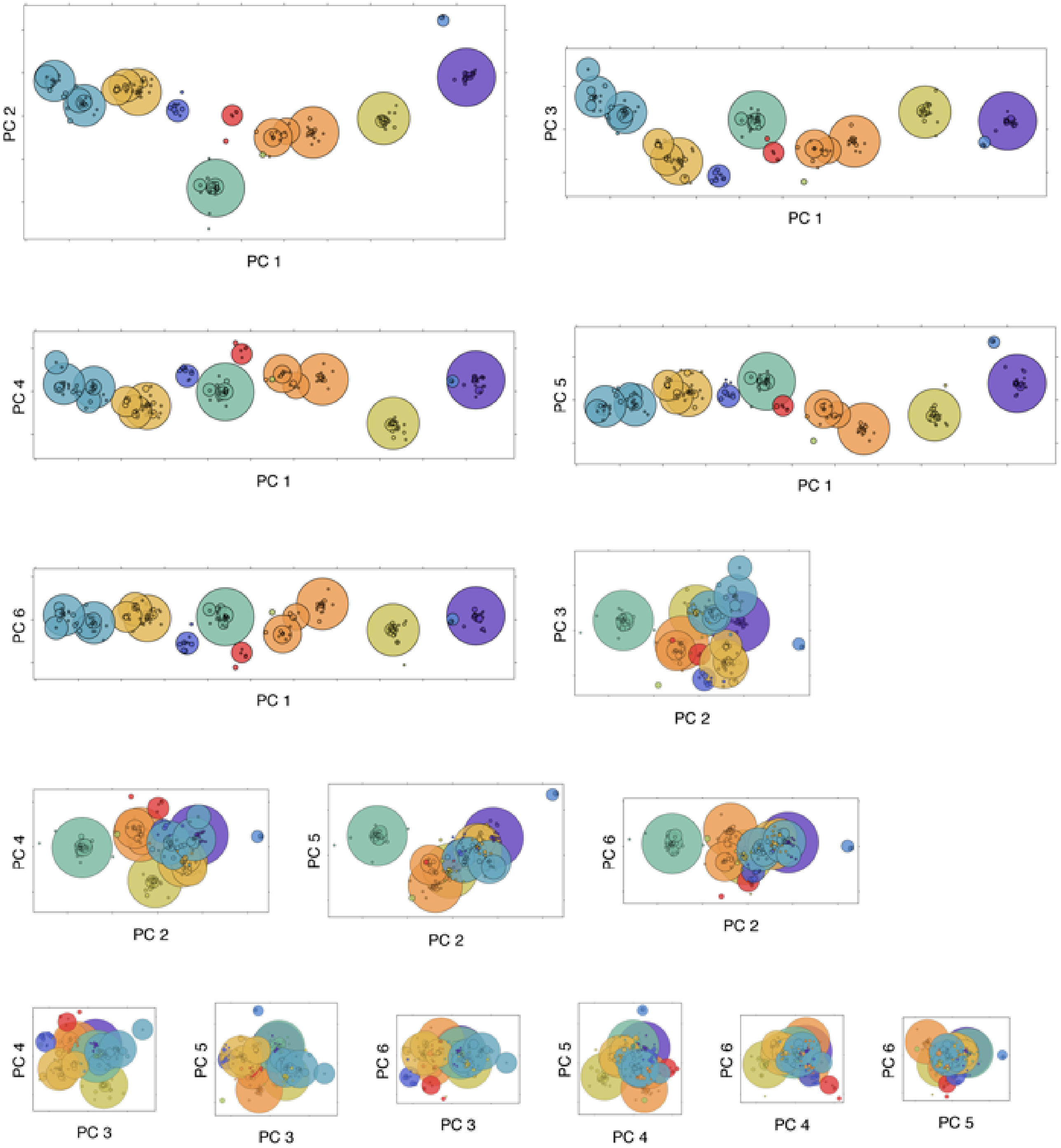

Figure S3. Principal components of antigenic variation under a 10-sphere mutation model. Each panel shows 5991 samples of antigenic phenotype over the course of a 40-year simulation. Each phenotype is represented as a bubble, with bubble area proportional to the number of samples with this phenotype. Bubbles are colored based on clustering the 10-dimensional antigenic phenotypes. The original 10-dimensional space was rotated using principal components analysis to give orthogonal axes in the order of their contribution to antigenic variation. Each panel shows a two-dimensional slice of the this rotated space. Principal components 7–10 were left out of the figure for clarity.
